# Large-scale observational study of AI-based patient and surgical material verification system in ophthalmology: real-world evaluation in 37 529 cases

**DOI:** 10.1136/bmjqs-2024-018018

**Published:** 2024-11-29

**Authors:** Hitoshi Tabuchi, Naofumi Ishitobi, Hodaka Deguchi, Yuta Nakaniida, Hayato Tanaka, Masahiro Akada, Mao Tanabe

**Affiliations:** 1 Department of Technology and Design Thinking for Medicine, Hiroshima University, Hiroshima, Japan; 2 Department of Ophthalmology, Tsukazaki Hospital, Himeji, Japan

**Keywords:** Patient safety, Medical error, measurement/epidemiology, Near miss, Surgery, Incident reporting

## Abstract

**Background:**

Surgical errors in ophthalmology can have devastating consequences. We developed an artificial intelligence (AI)-based surgical safety system to prevent errors in patient identification, surgical laterality and intraocular lens (IOL) selection. This study aimed to evaluate its effectiveness in real-world ophthalmic surgical settings.

**Methods:**

In this retrospective observational before-and-after implementation study, we analysed 37 529 ophthalmic surgeries (18 767 pre-implementation, 18 762 post implementation) performed at Tsukazaki Hospital, Japan, between 1 March 2019 and 31 March 2024. The AI system, integrated with the WHO surgical safety checklist, was implemented for patient identification, surgical laterality verification and IOL authentication.

**Results:**

Post implementation, five medical errors (0.027%) occurred, with four in non-authenticated cases (where the AI system was not fully implemented or properly used), compared with one (0.0053%) pre-implementation (p=0.125). Of the four non-authenticated errors, two were laterality errors during the initial implementation period and two were IOL implantation errors involving unlearned IOLs (7.3% of cases) due to delayed AI updates. The AI system identified 30 near misses (0.16%) post implementation, vs 9 (0.048%) pre-implementation (p=0.00067), surgical laterality errors/near misses occurred at 0.039% (7/18 762) and IOL recognition at 0.29% (28/9713). The system achieved>99% implementation after 3 months. Authentication performance metrics showed high efficiency: facial recognition (1.13 attempts, 11.8 s), surgical laterality (1.05 attempts, 3.10 s) and IOL recognition (1.15 attempts, 8.57 s). Cost–benefit analysis revealed potential benefits ranging from US$181 946.94 to US$2 769 129.12 in conservative and intermediate scenarios, respectively.

**Conclusions:**

The AI-based surgical safety system significantly increased near miss detection and showed potential economic benefits. However, errors in non-authenticated cases underscore the importance of consistent system use and integration with existing safety protocols. These findings emphasise that while AI can enhance surgical safety, its effectiveness depends on proper implementation and continuous refinement.

**WHAT IS ALREADY KNOWN ON THIS TOPIC**

- Artificial intelligence (AI) has shown promise in various medical applications, but its effectiveness in real-time surgical safety verification in ophthalmology was not well established.

**WHAT THIS STUDY ADDS**

- This large-scale study demonstrates that an AI-based surgical safety system can significantly increase near miss detection in ophthalmic surgeries (0.16% vs 0.048%, p<0.001). It quantifies the risk of intraocular lens errors (0.29%) and surgical laterality (0.039%) in a real-world setting. The study also highlights implementation challenges, including the importance of staff consensus and timely AI model updates.

**HOW THIS STUDY MIGHT AFFECT RESEARCH, PRACTICE OR POLICY**

- These findings may encourage wider adoption of AI-based safety systems in ophthalmic surgery and potentially other specialties by demonstrating AI’s effectiveness in simple yet critical tasks where human errors are common, such as patient identification and surgical site verification.

## Introduction

 Surgical errors in ophthalmology, though relatively rare, can have profound and life-altering consequences for patients.^1^ These errors, which include wrong-site surgery, incorrect intraocular lens (IOL) implantation and patient misidentification, not only compromise patient safety but also erode trust in healthcare systems, potentially affecting millions of patients globally due to the vast volume of cataract surgeries performed annually.^2^,^4^ A critical aspect of surgical safety in ophthalmology is surgical laterality, which refers to the correct identification and verification of the intended surgical site, specifically distinguishing between left and right sides of the body. This concept encompasses the processes of left-right recognition in preoperative planning and the prevention of wrong-side surgery, both of which are crucial for ensuring patient safety in ophthalmic and other surgical procedures. The WHO Surgical Safety Checklist, introduced in 2008, has reduced surgical errors across various specialties.^5 6^ However, its effectiveness in ophthalmic surgery—particularly for preventing laterality errors and incorrect IOL selection—is limited due to human error and workflow disruptions.^7^ Despite repeated safety alerts and reminders, these errors persist in ophthalmology.^7 8^

Advancements in artificial intelligence (AI) have shown promising results in medical fields such as diagnostic imaging and clinical decision support.^9 10^ While AI applications in healthcare safety exist for medication management^11^ and sepsis alerts,^12^ their use in real-time surgical safety verification, especially in ophthalmology, remains largely limited to our team’s pioneering work.^13 14^

Ophthalmology uniquely involves bilateral procedures and requires precise IOL selection, making it particularly vulnerable to laterality and implant-related errors.^7 15^ AI-based systems have been shown to enhance safety by verifying critical parameters such as patient identity, surgical site and IOL specifications in specific settings.^14^

However, the real-world implementation and effectiveness of such systems in ophthalmology have not been thoroughly evaluated, especially regarding integration into existing workflows, impact on error rates and cost-effectiveness.^16^,^18^ Questions remain about potential unintended consequences of introducing new technology into the complex environment of the operating room.^17^

To address these gaps, we developed and implemented an AI-based surgical safety system for ophthalmic procedures, integrated with the WHO Surgical Safety Checklist. Our study evaluates this approach in a real-world surgical setting over an extended period, analysing a large cohort of ophthalmic surgeries before and after implementation. We aimed to assess the system’s impact on surgical errors and near miss detection, identify implementation challenges and evaluate potential economic implications.

This study provides empirical evidence on the performance, limitations and economic impact of an AI-based surgical safety system, informing future strategies for improving patient safety in ophthalmic surgery and potentially other surgical specialties. Furthermore, a limited validation study conducted at another institution supports the efficacy and potential generalisability of our system.^14^

## Methods

### Study design and participants

This retrospective observational study compared ophthalmic surgeries before and after the implementation of an AI-based safety system at Tsukazaki Hospital, Japan. We included all patients undergoing ophthalmic surgery between 1 March 2019 and 31 March 2024 excluding the developmental period (1 April 2021 to 29 February 2022). The study comprised 18 767 surgeries in the pre-implementation period and 18 762 surgeries in the post implementation period.

### Data collection

Data for the pre-implementation period were extracted from the hospital’s medical safety reporting system and surgical records. For the post implementation period, data were collected from the AI-based surgery safety system logs, the hospital’s medical safety reporting system and surgical records.

### Incident reporting system

At Tsukazaki Hospital, all medical staff must verbally report incidents to their department heads (ophthalmology outpatient nurse, orthoptist, ophthalmic operating room staff or physician). The department head records the date, location and details electronically, ensuring the reporter’s anonymity by not including their name. They classify the incident as an error or near miss. Errors are reported to the medical safety committee, where the involved individual submits a written report. If necessary, the committee conducts an informal hearing to develop and implement preventive measures.

### Outcome measures

Primary outcomes were the number of medical errors (wrong patient, wrong eye or wrong IOL) and the number of near misses identified. Secondary outcomes included AI system implementation rate and authentication performance metrics (time taken, number of attempts). The surgical management system’s database structure and data entry process are illustrated in online supplemental figure 1. Comprehensive definitions and classification methods for errors and near misses are described in the following section, titled ‘Authentication process and outcome definitions’.

### AI-based surgery safety system

The AI-based surgery safety system integrates three main components: facial recognition, surgical laterality verification and IOL authentication. Online supplemental figure 2 provides an overview of these components. Our AI-based surgery safety system minimises additional workload by seamlessly integrating with existing hospital workflows and information systems. It uses patient demographic data from the hospital’s administrative database via secure network connections, eliminating duplicate data entry. Essential ophthalmic examination data are automatically retrieved from the structured ophthalmology surgical database. The only additional task is capturing the patient’s facial photograph for authentication, performed during routine preoperative patient education by nursing staff.

Postsurgical processes remain part of the standard workflow independent of the AI system, ensuring no significant additional administrative burden or workflow disruption. The minimal time required for facial photography is included in our cost calculations as part of nursing staff expenses.

The AI system’s interface uses a single iPad mini (Apple, USA) installed in each operating room, mounted on a charging station near the entrance when not in use. A dedicated circulating nurse manages the AI authentication process using this device, carrying it throughout the authentication sequence: facial recognition at the entrance, followed by surgical laterality and IOL authentication inside the operating room. The user interface is intuitive, with clear instructions and immediate feedback. Each authentication is initiated with a single tap, and the system provides real-time guidance for optimal image capture. Devices connect to the hospital network via secure Wi-Fi, enabling immediate data synchronisation and verification results. Detailed information about the system configuration, workflow integration and authentication processes is provided in online supplemental figure 1–3.

The system was designed to work in conjunction with the existing WHO Surgical Safety Checklist workflow, enhancing rather than replacing established safety protocols. Details of this integration process and the resulting workflow are provided in online supplemental figure 3.

Technical details of the system architecture and implementation specifications, including the use of YOLO V.3^18^ for surgical laterality verification and VGG V.16^19^ for IOL authentication, are provided in the online supplemental material: Detailed technical specifications and analyses, section 1: Technical details of AI components. Visual representations of the authentication processes and system workflows are illustrated in online supplemental figures 4 and 5, which provide detailed diagrams of the surgical laterality verification process and IOL authentication procedure, respectively.

Multiple IOLs were prepared for each surgery for two primary reasons: (1) to maintain sterility and provide backup in case of accidental contamination during the procedure, given the small size (approximately 6 mm) of the optical part and (2) to have alternative lens types available for unexpected intraoperative complications that might require different implantation techniques. This practice of preparing multiple IOLs influenced our AI system’s design, allowing for the authentication of stacked IOL packages.

### The capability of the AI-based surgery safety system

Pre-implementation testing with 300 cases (50% intentionally erroneous) demonstrated 100% system accuracy (online supplemental figure 6).

### Authentication process and outcome definitions

Each of the three authentication types (facial, surgical laterality, IOL) has defined quality standards for authentication photos. If the image quality does not meet these standards, an alert screen prompts for reshooting (online supplemental figure 7). The authentication process follows a defined flow with specific outcomes:

1. Authentication not performed: cases where authentication is not attempted.
2. Authentication successful: the authentication photo meets quality standards and AI determines it to be correct.
3. Near miss: AI incorrectly identifies a case, but the error is corrected before surgery.
4. Error: surgery proceeds incorrectly, either without authentication or with an uncorrected AI misidentification.
5. Authentication failure: multiple authentication attempts do not meet quality standards, but surgery proceeds at the surgeon’s discretion. The primary causes of authentication failures were human factors in image capture, such as incorrect camera angles, poor focus, inadequate lighting or obstructions in the field of view, rather than hardware limitations. Online supplemental figure 8 provides representative examples of these authentication failures due to poor image quality.

Online supplemental figure 9 illustrates this authentication process and the defined outcomes.

### Data management and quality control

The system’s efficacy depends on accurate initial data input,^20 21^ with detailed quality control processes documented in online supplemental figure 1, 10 and 11. The IOL recognition model was updated three times during the study period (29 March 2022, 2 August 2022 and 25 January 2023) to maintain compatibility with new IOL models.

### Cost–benefit and cost-effectiveness analyses

To evaluate the economic impact of the AI-based surgery safety system, we conducted both cost–benefit analysis (CBA) and cost-effectiveness analysis (CEA) over a projected 5-year period. Our analyses encompassed system implementation costs (hardware, software development, AI model training and implementation), operational costs (maintenance, additional personnel, training) and compared these against the costs of traditional safety measures.

For the CBA, benefits were quantified by calculating costs related to medical errors, including additional treatment expenses, estimated legal compensation and potential revenue impact due to hospital reputation decline.^22^,^24^ The number of prevented medical errors was considered as the key outcome measure. All future costs and benefits were discounted to present value using a 3% annual rate, standard in healthcare economic evaluations.^25 26^

In our economic evaluation model, we considered the potential impact of near misses detected by the AI system. While these near misses did not result in actual errors in our study due to AI implementation, we modelled scenarios where they might have progressed to errors without the AI system. We used three probability scenarios (0%, 50% and 100%) for near misses becoming errors, representing conservative, intermediate and high-risk estimates respectively. This approach allows us to estimate a range of potential economic benefits across different risk levels.

For both CBA and CEA, we developed three scenarios to capture the range of possible outcomes: a conservative setting (assuming prevention of only observed errors), an intermediate setting (assuming 50% of near misses would have resulted in errors) and a high-risk setting (assuming all near misses would have resulted in errors).

The CEA used the incremental cost-effectiveness ratio (ICER), calculated as:

ICER = (Cost_AI − Cost_traditional)/(Effect_AI − Effect_traditional)

where Cost_AI and Cost_traditional represent the total costs of the AI system and traditional safety measures, respectively, and Effect_AI and Effect_traditional represent the number of errors prevented by each approach. We assumed that traditional safety measures prevented one error during the study period.

For the economic evaluation, we used US healthcare cost data despite the study being conducted in Japan. This decision was made to enhance international comparability, leverage the comprehensive nature of US healthcare cost data and provide a conservative estimate of potential economic benefits. We acknowledge that this approach may limit the direct applicability of our economic findings to the Japanese healthcare system. To address this limitation, we conducted sensitivity analyses to account for potential variations in healthcare costs across different systems.

To assess the robustness of our findings, we performed sensitivity analyses^27^ by varying key parameters such as the rate of medical errors and the amount of legal compensation by ±20%. Furthermore, to quantify uncertainty, we conducted a Monte Carlo simulation^28^ with 10 000 iterations, establishing 95% CIs for our cost estimates.

All costs were calculated in US dollars. A comprehensive description of the cost and benefit calculation methods is available in online supplemental table 1.

### Related studies

Prior to our large-scale study, a limited validation study of a similar AI-based surgical safety system was conducted at Tsukuba University Hospital between February and May 2022. This study evaluated the system’s performance in 171 patients (171 eyes) undergoing phacoemulsification and IOL implantation.^14^

## Results

### Participant characteristics

During the study period, we analysed 37 529 ophthalmic surgeries: 18 767 in the pre-implementation period (March 2019 to February 2022) and 18 762 in the post implementation period (March 2022 to March 2024). Table 1 presents the demographic and surgical characteristics of the participants. Notably, patients in the pre-implementation period were significantly older than those in the post implementation period (mean age (SD): 69.2 (15.7) years vs 67.6 (16.4) years, p<0.001).

**Table 1:** Demographic and clinical characteristics of patients in pre-implementation and post implementation periods

Characteristic	Pre-implementation Period	Post implementation Period	P value
Study period	1 March 2019 to 31 March 2021	1 March 2022 to 31 March 2024	
Number of surgeries	18 767	18 762	
Number of patients	8823	8659	
Age, mean (SD)	69.2 (15.7)	67.6 (16.4)	<0.001*
Female, n (%)	4427 (50.2%)	4412 (51.0%)	0.304†
IOL implantation, n (%)	9644 (51.4%)	9713 (51.8%)	0.463†
General anaesthesia, n (%)	752 (4.0%)	692 (3.7%)	0.013†

* Wilcoxon test.

† Fisher’s exact test.

IOL, intraocular lens.

### Primary outcomes

#### Medical errors

In the post implementation period, we observed five medical errors (0.027%, 95% CI: 0.009 to 0.062%) out of 18 762 surgeries. Four of these errors occurred in cases without AI authentication. Comparatively, the pre-implementation period saw one reported case of incorrect IOL insertion (0.0053%, 95% CI: 0.0001% to 0.030%, 1/18,767). The OR for medical errors in the post implementation period compared with pre-implementation was 5.00 (95% CI: 0.58 to 42.80, p=0.125), indicating no statistically significant difference (table 2). Despite multiple safety checks, including three patient confirmations (during consultation, entry to the OR and anaesthesia), five errors occurred post implementation. Two involved incorrect IOL implants, which lacked patient confirmation. The other three were wrong-side errors: one Botox injection on the wrong eye bypassed both the timeout and AI authentication, while in the other two, surgical drapes were misapplied after verbal confirmation, with injections proceeding either without AI authentication or amid AI detection, where a nurse hesitated to alert the surgeon. All five cases reportedly had certain safety protocols in place, yet these measures failed to prevent the errors. The error process was documented with images of the AI identification and is shown in online supplemental figure 12.

**Table 2:** List of error cases

Case	Occurrence	Event	AI-based surgery safety	WHO Surgical Safety Checklist	Error details and outcome
Case 1	May 2023	Steroid injection for wrong eye.	Not implemented	Implemented	A timeout check involving three staff was conducted, but they did not notice that the surgical drape was incorrectly placed, leading to an injection in the wrong eye. The patient was observed without treatment, and no sequelae occurred.
Case 2	May 2023	Botox injection for wrong eye.	Not implemented	Not implemented	A nurse encouraged the doctor to perform a timeout check, but the doctor refused, resulting in an injection in the wrong eye. The patient was observed without treatment, and no sequelae occurred.
Case 3	September 2023	Local anaesthetic injection for wrong eye.	Implemented	Implemented	A timeout check involving four staff was conducted, but none of them noticed the incorrect placement of the surgical drape. The AI-based surgery safety system continued to point out the mistake, but the attending nurse could not relay this to the surgeon even after eight attempts, leading to a local anaesthetic injection in the wrong eye. The patient was observed without treatment, and no sequelae occurred.
Case 4	March 2024	Wrong IOL implantation	Not implemented	Implemented	A timeout check involving three staff was conducted, but none of them noticed the wrong IOL had been prepared for a different patient, which was then implanted. The correct IOL was implanted the following day, and no sequelae occurred.
Case 5	March 2024	Wrong IOL implantation	Not implemented	Implemented	A timeout check involving three staff was conducted, but none of them noticed the wrong IOL had been prepared for a different patient, which was then implanted. The correct IOL was implanted the following day, and no sequelae occurred.

AI, artificial intelligence; IOL, intraocular lens.

#### Near misses

**Figure 1:**
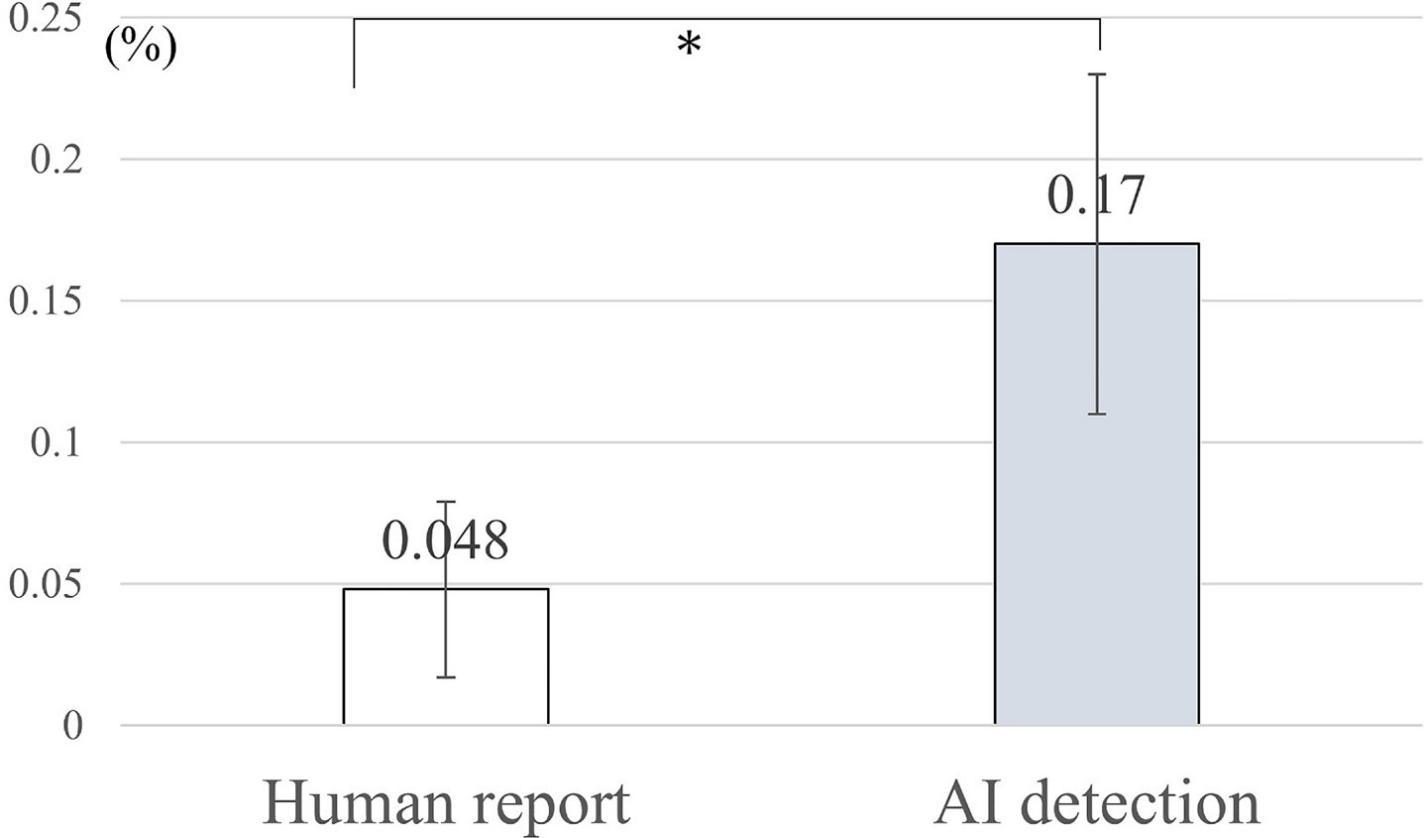
Comparison of near miss incident rates: human reporting versus artificial intelligence (AI) detection. This bar chart compares the near miss incident rates detected by human reporting and AI detection in ophthalmic surgeries. The y-axis represents the incident rate as a percentage, while the x-axis shows the two detection methods. The black bar represents human-reported incidents (0.048%), while the grey bar represents AI-detected incidents (0.17%). Error bars indicate the 95% CI. The graph demonstrates that AI detection identified a significantly higher rate of near miss incidents compared with traditional human reporting methods, (*p=0.00067 by Fisher’s exact test) suggesting improved sensitivity in identifying potential safety issues.

The AI system identified 30 near misses (0.16%, 95% CI: 0.11% to 0.23%) in the post implementation period, including four cases of incorrect left-right drape placement and 26 cases of IOL preparation mistakes. In contrast, nine near misses (0.048%, 95% CI: 0.022% to 0.091%) were reported in the pre-implementation period through the hospital’s incident reporting system. The OR for near miss detection in the post implementation period was 3.34 (95% CI: 1.59 to 7.01, p=0.00067), indicating a significant improvement in detection (figure 1). Representative examples of both error types are shown in online supplemental figure 13.

#### Error and near miss rates by authentication type

**Figure 2:**
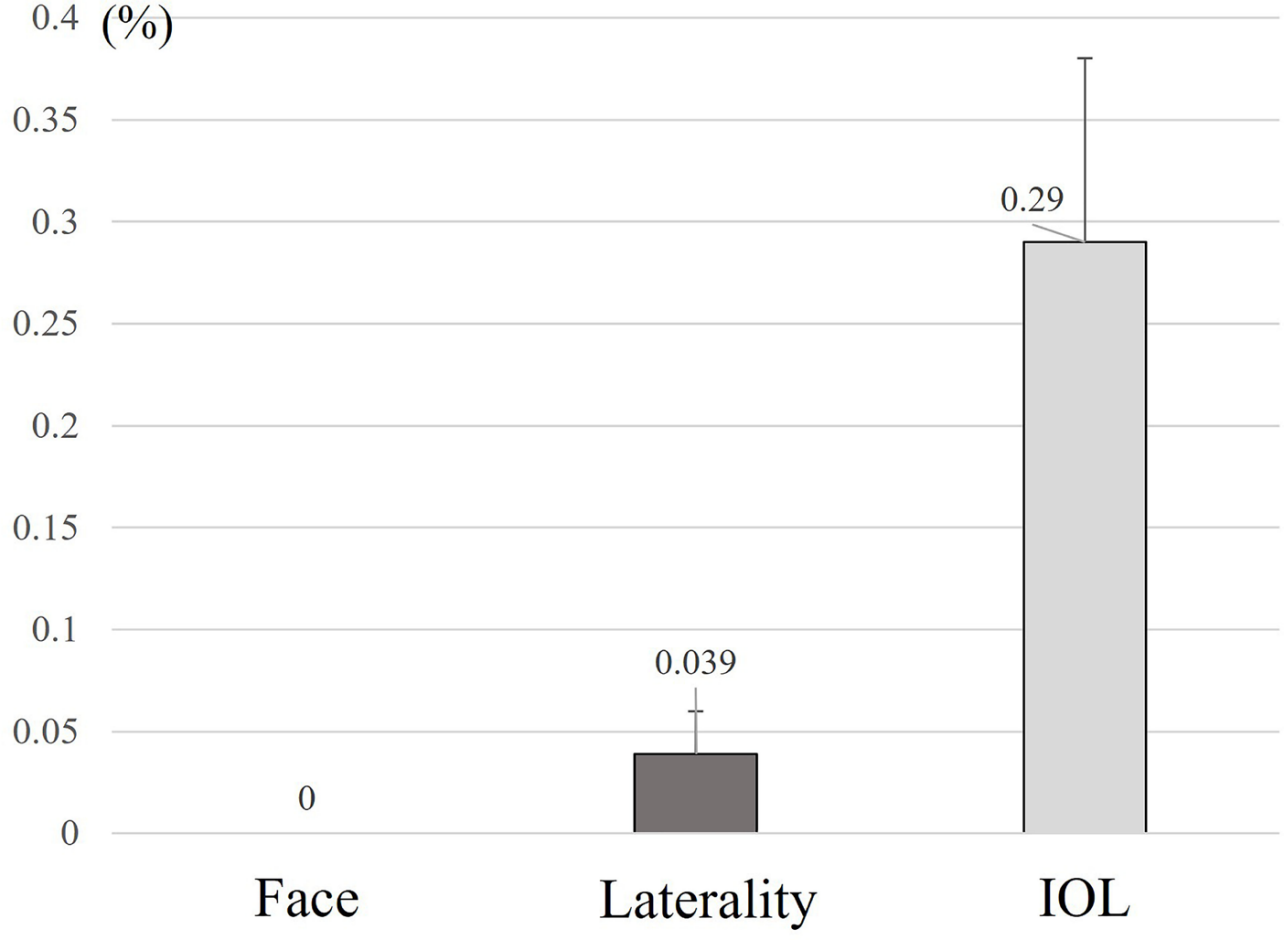
Error and near miss rates in each of the three authentications. Face: No errors or near misses were identified through facial recognition, resulting in an occurrence rate of 0%. Left-right: There were seven errors or near misses related to surgical laterality, resulting in an occurrence rate of 0.039% (95% CI: 0.018% to 0.077%). IOL, intraocular lens. There were 28 errors or near misses related to IOL recognition, resulting in an occurrence rate of 0.29% (95% CI: 0.20% to 0.42%). Error bars represent the upper bound of the 95% CI.

Figure 2 illustrates the error and near miss rates for each type of authentication detected by the AI-based safety system. There were seven errors or near misses in surgical laterality, with an occurrence rate of 0.039% (95% CI: 0.018% to 0.077%) out of 18 762 opportunities. For IOL recognition, there were 28 errors or near misses, resulting in an occurrence rate of 0.29% (95% CI: 0.20% to 0.42%) out of 9713 opportunities. Online supplemental table 2 shows the previously described numbers for each outcome definition, along with a diagram.

### Secondary outcomes

#### AI system implementation

**Figure 3:**
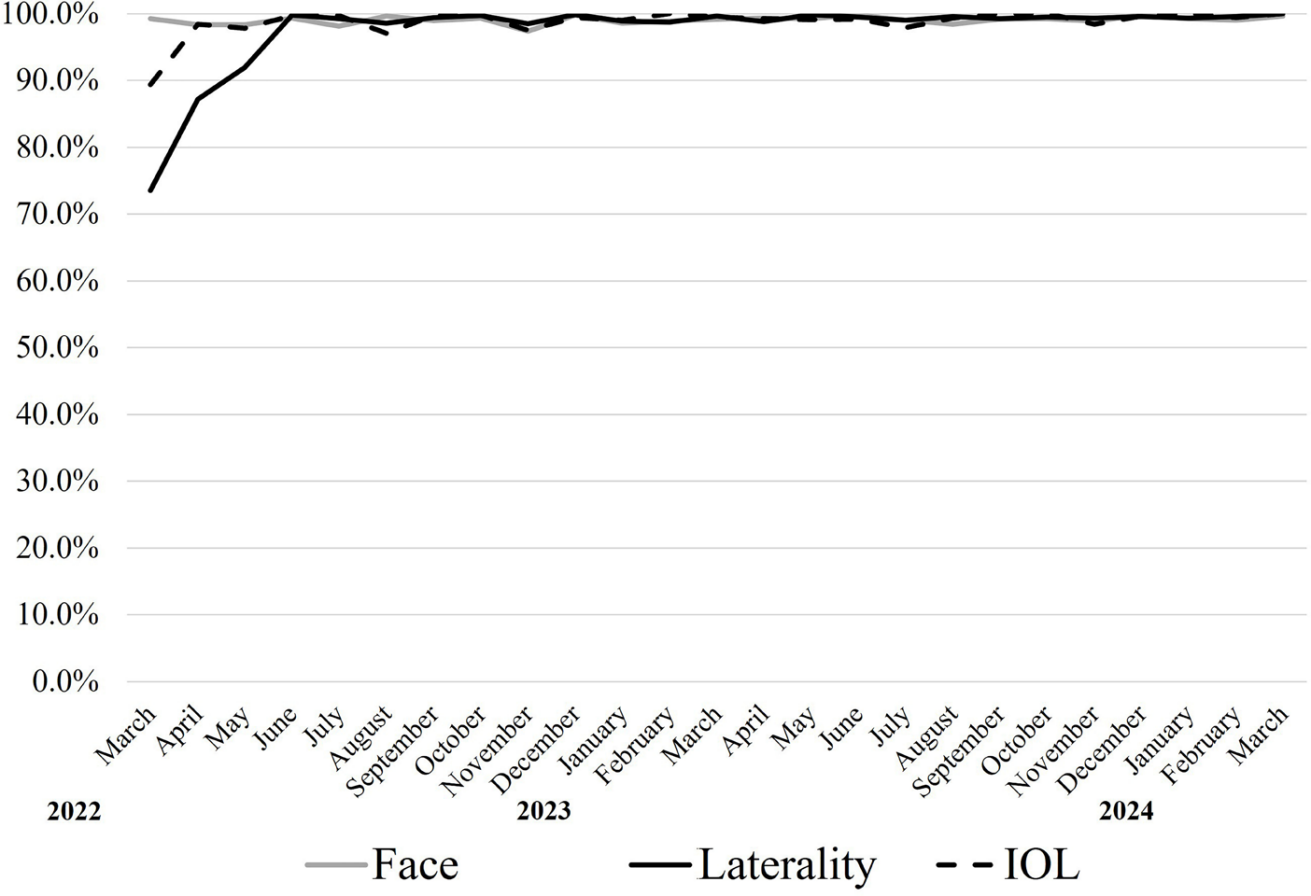
Trend of artificial intelligence (AI)-based surgery safety authentication rates. Trends in the authentication rates for AI-based surgery safety among eligible cases were plotted. The grey solid line indicates facial recognition, the black solid line indicates surgical laterality and the black dashed line indicates intraocular lens (IOL) recognition. It took 3 months for the implementation rate of AI-based surgery safety to exceed 99% for all three types of recognition. During the initial implementation phase, two laterality errors occurred among the unperformed cases.

After a 3-month implementation period, the AI system achieved an implementation rate exceeding 99% for all three authentication types—facial recognition, surgical laterality and IOL authentication—as illustrated in figure 3.

### Unlearned IOLs and authentication failures

Of the 9713 IOL surgeries performed post implementation, 7.3% involved unlearned lenses, leading to two cases of incorrect IOL implantation. Authentication failures due to substandard image quality were low but notable, occurring in 0.25% of facial recognition cases, 0.24% of surgical laterality cases and 0.66% of IOL recognition cases. These findings underscore the critical importance of image quality in the authentication process and highlight areas for potential improvement in system usability.

Detailed performance metrics, including the mean number of attempts and time required for each recognition type, are provided in the online supplemental material: Detailed technical specifications and analyses under performance metrics details.

### Implementation challenges

During the initial 3-month implementation period, we observed lower authentication rates, particularly for surgical laterality (84.5% vs 99.4% post implementation). This period saw two laterality errors, highlighting the importance of rapid consensus-building among staff for consistent system use.

Online supplemental table 3 provides details on these previously described implementation rates and ineligible cases.

### Economic evaluation

Our economic evaluation, which included both CBA and CEA, indicated potential economic advantages of the AI-based surgery safety system over traditional safety measures. Over a 5-year period, the total cost of implementing the AI system was US$461 426.49, compared with US$125 872.46 for traditional methods.

In the intermediate risk setting—assuming that 50% of near misses would have resulted in errors—the AI system demonstrated a positive net present value (NPV) of US$2 093 468.31 (95% CI: US$1 519 215.03 to US$2 667 721.59). The ICER in this scenario was US$18 641.89 per additional error prevented.

The sensitivity analysis indicates that even with fluctuations in error rates, the AI system maintains a positive NPV, confirming its economic viability across various scenarios. Detailed economic analyses, including results from conservative and high-risk scenarios as well as sensitivity analyses, are provided in the online supplemental material: Detailed technical specifications and analyses under detailed economic analyses.

### Related studies

A prior validation study at Tsukuba University Hospital^14^ reported first-attempt authentication rates of 92.0% for facial recognition, 82.5% for laterality confirmation and 67.4% for IOL parameter verification. After repeated attempts, these rates improved to 96.3%, 98.2% and 88.9%, respectively. Once authentication was successful, both false rejection and false acceptance rates were 0% for all parameters, consistent with our findings.

## Discussion

Our large-scale retrospective study of an AI-based surgery safety system in ophthalmic surgeries revealed critical findings with significant implications for patient safety. Near miss detection increased significantly after system implementation (0.16% vs 0.048%, p<0.001), demonstrating the enhanced sensitivity of AI-assisted safety protocols over traditional reporting methods and addressing the under-reporting challenge in healthcare safety.^29 30^

The AI system detected an IOL error risk of 0.29% and a surgical laterality risk of 0.039%. Extrapolated to the approximately 1.3 million cataract surgeries performed annually in Japan,^3^ this suggests up to 3770 cases may be at risk for IOL implantation errors each year, underscoring the need for enhanced safety measures in high-volume ophthalmic procedures.

The system’s high implementation rate (>99% after 3 months) indicates good acceptability among surgical staff. However, the initial learning curve and resistance highlight the importance of thorough training and change management strategies when adopting new technologies.^17^ The discrepancy between pre-implementation and post implementation error reporting (1 vs 5 cases) likely reflects improved detection rather than an actual increase in errors, emphasising the need to consider both quantitative and qualitative factors when evaluating safety systems. For example, online supplemental figure 12 shows a case where a physician administered anaesthesia to the wrong eye—an error not recognised at the time—illustrating the system’s capability for detailed post hoc analysis beyond standard human reporting.

The observed increase in reported errors post implementation highlights reporting bias in healthcare safety research. Traditional reporting systems significantly underreport errors and near misses^31^ due to factors like fear of repercussions, lack of time or failure to recognise reportable incidents.^32^ By objectively detecting and logging incidents, our AI-based system likely provides a more accurate error rate. This difference underscores challenges in directly comparing pre-implementation and post implementation error rates and suggests the baseline error rate may have been higher than reported, further emphasising the benefits of AI-assisted protocols. Future research should consider methods to estimate and account for this reporting bias, such as capture–recapture techniques or qualitative studies of staff reporting behaviours.^33 34^

Our CBA showed potential economic benefits ranging from US$181 946.94 in the most conservative setting to US$2 769 129.12 in a more realistic intermediate scenario. These findings suggest the system enhances patient safety and offers significant economic advantages, though these projections are based on US cost data. Although our study was conducted in Japan, we used US cost data for the economic evaluation to enhance international relevance and provide a conservative estimate, given the higher healthcare costs in the USA. This choice limits the direct applicability of our economic results to the Japanese healthcare system or other national contexts, as the actual economic impact may vary depending on specific healthcare systems and cost structures. To address this limitation, we conducted sensitivity analyses accounting for potential variations in healthcare costs, which indicated that the economic benefits remain substantial across different scenarios. We recommend that future research perform similar economic evaluations using cost data from multiple countries to provide a more comprehensive global perspective on the economic impact of AI-based safety systems, thereby supporting informed decision-making regarding their implementation in diverse healthcare environments.

The occurrence of errors, particularly in cases without AI authentication, underscores the importance of consistent system use and integration with existing safety protocols. The case where an error occurred despite both WHO checklist and AI authentication use highlights the complex interplay between technology and human factors in surgical safety, emphasising the need for clear communication protocols and a culture of psychological safety in ophthalmic operating rooms.^35^ The persistence of errors despite multiple safety checks highlights the complex challenges in surgical safety. Our findings underscore the limitations of current manual verification methods, even when rigorously applied. The two IOL implantation errors, which went undetected despite a timeout procedure involving one physician and two nurses, exemplify the potential shortcomings of existing protocols. This aligns with previous reports on the inconsistency of timeout procedures and the variable engagement of medical staff in safety checks.

Our observations suggest that while verbal confirmation and timeout procedures are crucial, they are not infallible. Human factors, such as distraction, fatigue or interpersonal dynamics in the operating room, can compromise the effectiveness of these safety measures. The case where a physician disregarded both the timeout procedure and AI authentication particularly illustrates how individual actions can circumvent established safety protocols.

The integration of AI authentication as an additional layer of safety could potentially mitigate these risks. By providing an objective, consistent verification mechanism that is less susceptible to human factors, AI authentication could complement existing safety procedures. Our data suggest that achieving 100% implementation of AI authentication could further reduce errors. This technological implementation, combined with ongoing efforts to strengthen safety culture and improve adherence to existing protocols, presents a promising approach to enhancing surgical safety in ophthalmology.

The issue of unlearned IOLs in 7.3% of cases highlights a significant challenge in keeping AI systems relevant in the rapidly evolving field of ophthalmic surgery. This underscores the importance of adaptive AI models and continuous updates to the system, as understood in the context of known machine learning operations.^36^ System updates were conducted three times during the study period. While more frequent updates could potentially improve system performance, particularly in recognising new IOL models, the update frequency was constrained by both budgetary limitations and the need to balance system stability with the introduction of new features. Future iterations of the system will aim to implement a more dynamic update process to address this limitation.

Despite strengths such as a large sample size and real-world setting, our study is limited by its single-centre design and retrospective nature. However, the retrospective approach was crucial for comprehensive error analysis, allowing detailed post hoc examination of incidents not recognised as errors at the time.

A smaller validation study at Tsukuba University Hospital^14^ showed similar authentication rates and perfect postauthentication accuracy, supporting our findings. This cross-institutional validation suggests our AI-based system may be effective in different healthcare environments, though larger multicentre studies are needed to fully establish its generalisability.

In conclusion, our AI-based surgery safety system shows significant promise in enhancing near miss detection in ophthalmic surgeries, revealing alarming potential error rates in high-volume procedures. These findings highlight the urgent need for a comprehensive approach to patient safety that combines technological solutions with improved reporting cultures and continuous quality improvement efforts. Future research should focus on multicentre prospective studies, cost-effectiveness analyses and developing adaptive AI models to address evolving surgical techniques and equipment. While there is promising evidence for the system’s applicability across different healthcare environments, larger multicentre studies encompassing a wider range of ophthalmic procedures are necessary to confirm its generalisability and long-term impact on patient outcomes and healthcare provider behaviour.

## Supplementary material

10.1136/bmjqs-2024-018018 online supplemental file 1

## Data Availability

Data are available on reasonable request.
