# Supplementary material for: Large-scale observational study of AI-based patient and surgical material verification system in ophthalmology: real-world evaluation in 37 529 cases
Source: BMJ Qual Saf. 2024 Nov 29;34(7):e018018. doi: 10.1136/bmjqs-2024-018018 (PMC12229072; doi:10.1136/bmjqs-2024-018018)
Supplement: online supplemental file 1 [file bmjqs-34-7-s001.pdf]

Supplemental Material

Contents

Supplemental Figures

- Figure 1: The AI-based Surgery Safety system consists of three main components
- Figure 2: Integration of AI-based Surgery Safety System with Patient Care Workflow and Hospital Information Systems
- Figure 3: Integration of the AI-based Surgery Safety system with the WHO Surgical Safety Checklist workflow
- Figure 4: The process of laterality verification and the allowed photographing range
- Figure 5: The IOL authentication process and the variations in IOL package printing
- Figure 6: AI System Performance in Authentication Tasks: 100% Sensitivity and Specificity
- Figure 7: Alert Screen Prompting Re-Shooting Due to Low Quality of Authentication Image
- Figure 8: Examples of Authentication Failures Due to Poor Quality of Authentication Photos
- Figure 9: Authentication process and definition of terms
- Figure 10: The process of IOL data entry into the reference database
- Figure 11: The data entry and verification process
- Figure 12: Sequence of AI Authentication Attempts in a Case of Wrong-Side Anesthesia Administration
- Figure 13: AI Authentication Process in Preventing Intraocular Lens and Surgical Draping Errors
- Figure 14: Monthly Trend of IOL Authentication Rate and Unlearned IOL Usage Ratio

Supplemental Tables

- Table 1: Calculation process of cost benefit analysis
- Table 2: The number for each outcome definition
- Table 3: Implementation rates and ineligible cases

Detailed Technical Specifications and Analyses

1. Technical Details of AI Components
   - Facial Recognition System
   - Surgical Laterality Verification
   - IOL Authentication System
2. Performance Metrics Details
   - Authentication Attempts and Time Analysis
   - Unlearned IOLs Incidence of Unlearned Lenses

-Authentication Failures Due to Substandard Image Quality
3. Detailed Economic Analyses
- Net Present Value (NPV) Calculations
- Cost-Effectiveness Analysis (CEA)
- Sensitivity Analysis
- Summary of Economic Findings

Supplemental Figure 1

The AI-based Surgery Safety system consists of three main components

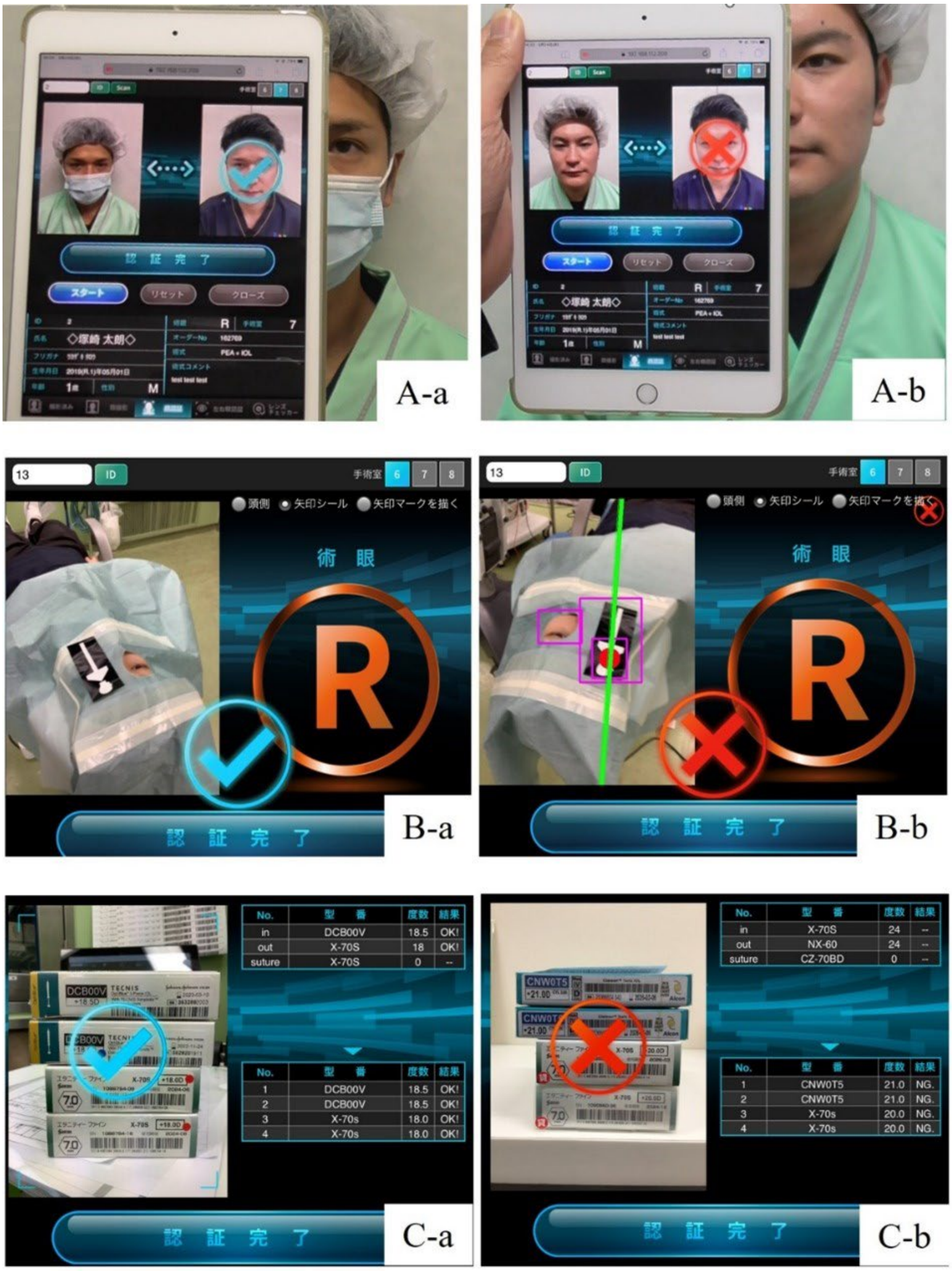

A: Facial Recognition Photo A-a: When the correct patient is identified, a blue checkmark is displayed

on the reference face photo. A-b: When the wrong patient is identified, a red cross mark is displayed on the reference face photo. B: Surgical laterality Photo B-a: When the correct side is identified, a blue checkmark is displayed in the center. B-b: When the wrong side is identified, a red cross mark is displayed in the center. C: Intraocular Lens (IOL) Recognition Photo C-a: When the correct IOL set is identified, a blue checkmark is displayed on the lens photo. C-b: When the wrong IOL is identified, a red cross mark is displayed in the center.

Supplemental Figure 2

Integration of AI-based Surgery Safety System with Patient Care Workflow and Hospital Information Systems

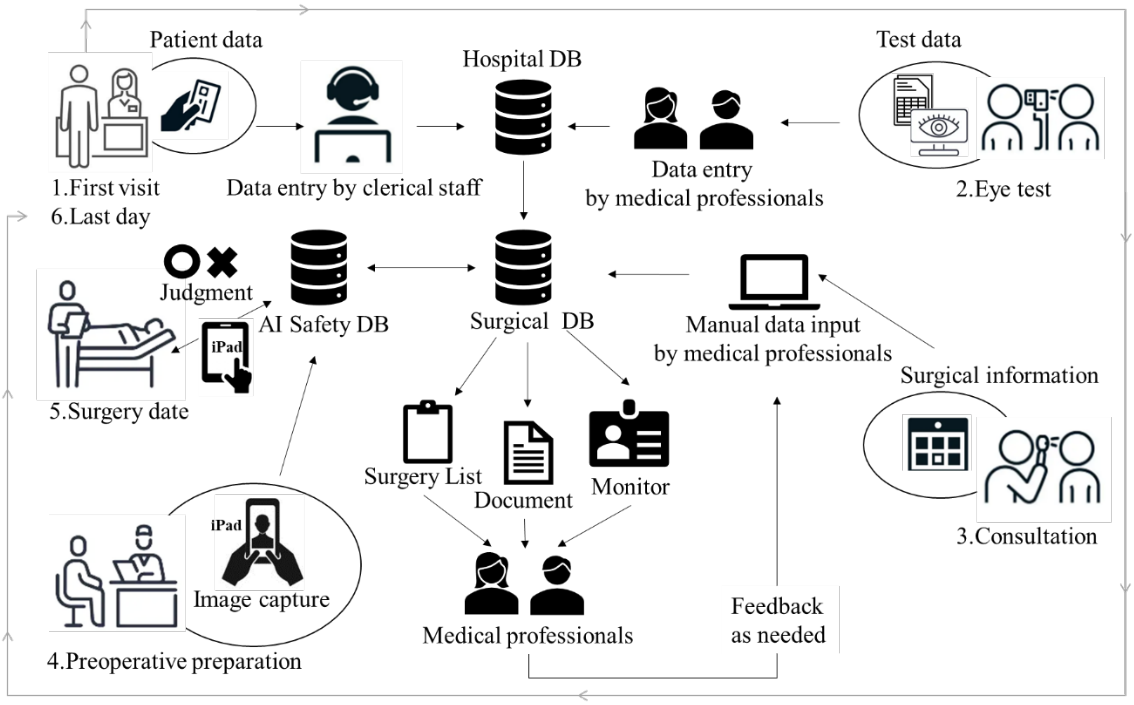

This diagram illustrates the comprehensive integration of the AI-based Surgery Safety system within both the patient care pathway and hospital information infrastructure. The outer circle (white pictograms) represents the patient journey from first visit through discharge (steps 1-6), while the inner components show the data flow and verification processes among various hospital systems.

Patient data, initially collected during registration (step 1) and eye examination (step 2), is entered into the hospital database by clerical staff and medical professionals. During the surgical consultation (step 3), the surgical plan is documented and automatically integrated into the system. The only additional data collection specifically required for the AI Safety system occurs during preoperative preparation (step 4), where a facial photograph is captured for authentication purposes.

The central portion shows how data flows between the hospital database, surgical database, and AI Safety database, with multiple verification points by medical professionals. This integrated approach

eliminates duplicate data entry and ensures that all necessary information is seamlessly shared across systems. The AI system provides real-time safety checks during surgery (step 5), with the process concluding at the final visit (step 6).

Supplemental Figure 3

Integration of the AI-based Surgery Safety system with the WHO Surgical Safety Checklist workflow.

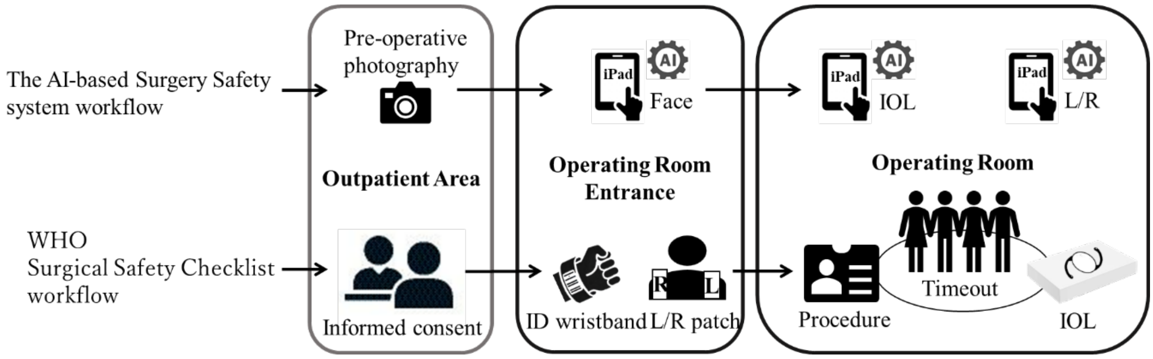

This diagram illustrates the parallel processes of the AI system and traditional safety checks from the outpatient area through the operating room. The authentication applications for the three types of recognition (Facial, IOL, Left/Right) are installed on iPads in each of the three ophthalmic surgery rooms. The reference photos for facial recognition are taken in the ophthalmology outpatient clinic before surgery and stored on the reference server. The AI model on the AI server verifies whether the authentication photos taken with the iPads match the surgical information master data obtained via the reference server.

Pre-operative facial photographs are taken by the nurse responsible for patient education, which occurs simultaneously with the surgeon's explanation of the procedure. This workflow helps mitigate the risk of photographing the wrong patient. The application of wristbands and patches indicating the surgical eye on the patient's shoulders is performed at the operating room entrance, prior to AI facial recognition.

Within the operating room, after AI-based laterality and IOL authentication are completed, a timeout procedure is conducted with all operating room staff present. This involves a visual confirmation of the checklist items, further enhancing patient safety through multiple layers of verification

Supplemental Figure 4

The process of laterality verification and the allowed photographing range

The right-left authentication model was built with a two-stage object detection model (You Only Look Once (YOLO) v3) [1] and identifies the positional relationship between the opening on the surgical drape placed over the operative eye and an arrow sticker indicating the nonoperative side

for authentication. It was necessary to apply two labels, the direction of the arrow and the relationships between the arrow and the opening, so we separated the process into two. In the first stage, the model identifies the arrowhead of the arrow sticker as the upwards direction. In the second stage, the model identifies the operative side as right or left based on the positional relationship between the direction-defined arrow sticker and the opening of the drape. A sample photograph depicting the configuration of the drape is shown in A. The system defines the drape opening as on the left or right based on the midline with the head direction detected by the AI identification using the arrow axis and arrowhead. This method relies on the knowledge that individuals are far less likely to demonstrate cognitive errors with regard to the top-bottom and back-front axes as they do with regard to the left-right axis in three-dimensional space. [2] Therefore, the arrow seal must always be explicitly placed on the midline side rather than the opening with the correct top-bottom axis. In the implementation phase, the staff was instructed to place the arrow over the opposite eye with the arrowhead pointed towards the top of the patient's head. We collected the model training dataset photographs in September 2021 as follows. First, a Tsukazaki Hospital operating room staff member lay on a surgical bed and was draped, and an arrow sticker was placed over the nonoperative eye with the arrowhead pointing toward the head. Then, photographs that clearly depicted the opening of the surgical drape and the arrow sticker indicating the right or left side were taken with an iPad mini at random positions within the areas indicated in red in B and C, which are the only areas where such left-right authentication photographs are allowed to be taken. We set the range to include all the photographer's standing positions to capture the aperture in a natural posture. Therefore, we did not explain this shooting range to medical staff during the certification process. A total of 1,171 images were used to train the YOLO model for the first stage (momentum=0.9, decay=0.000001, learning rate=0.001), and 1,167 images were used to train the model for the second stage (momentum=0.9, decay=0.000001, learning rate=0.001).

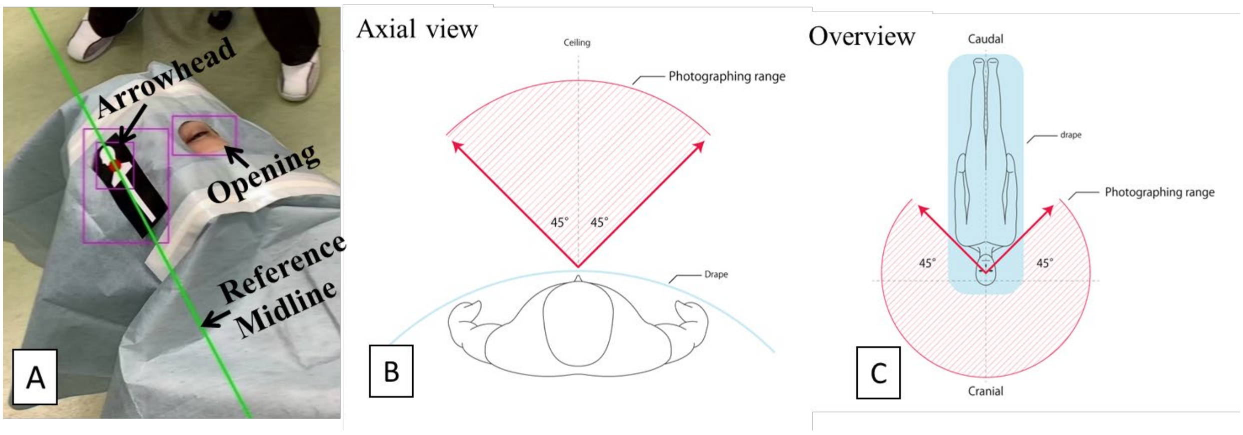

A, An R-L authentication photo superimposed with bounding boxes indicating the opening of drape

and the arrow sticker, a red dot indicating the position of the arrowhead (pointing to the patient's head), and a straight green line that serves as a reference midline. The system determines left or right according to whether the AI-identified opening is on the left or right of the AI-identified reference midline. B, C: Areas where the R-L authentication photographs can be taken. B, on axial view, the training images can be taken within a range of 45 degrees from directly above the patient. C, on overview, the training images can be taken within a range of 270 degrees, excluding 90 degrees in the foot direction.

Supplemental Figure 5

The IOL authentication process and the variations in IOL package printing

The IOL authentication model reads the authentication areas on the IOL package that module must read to extract information on the lenses. This model can identify up to six stacked IOL boxes, as shown in A. The IOL authentication model was divided into three stages: identification of the authentication area by another YOLOv3, identification of the IOL model number by VGG16 [3], and identification of the IOL power by yet another VGG16. We performed IOL certification by stacking the outer boxes of the IOLs and photographing their short sides, on which the label that contains all the information to be authenticated is printed as shown in B. Up to six packs of IOLs can be authenticated simultaneously without removing the sterile seal packaging. The iPad mini displays the IOL data from the reference database and the result on the authentication screen. (Supplemental Figure 1C-a and C-b) As of March 20th, 2022, the authentication area YOLOv3 has been trained with 1,732 images (momentum=0.9, decay=0.0005, learning rate=0.001); the IOL model number VGG16 has been trained with 123,616 IOL model number images, covering 136 IOL models (augmented from an initial 30,966 images; 82,548 for training and 41,068 for validation; momentum=0.001, decay=0.000001, learning rate=0.001); and the IOL power VGG16 has been trained with 57,126 IOL power identification images, covering IOLs with 121 different powers ranging from -6.0 to +30.0 spherical dioptres in 0.5 dioptre increments and cylinder dioptres of each toric IOL model (augmented from an initial 19,317 images; 45,802 for training and 11,324 for validation; momentum=0.001, decay=0.000001, learning rate=0.001). All images for the study were acquired by photographing unused IOL boxes with an iPad mini.

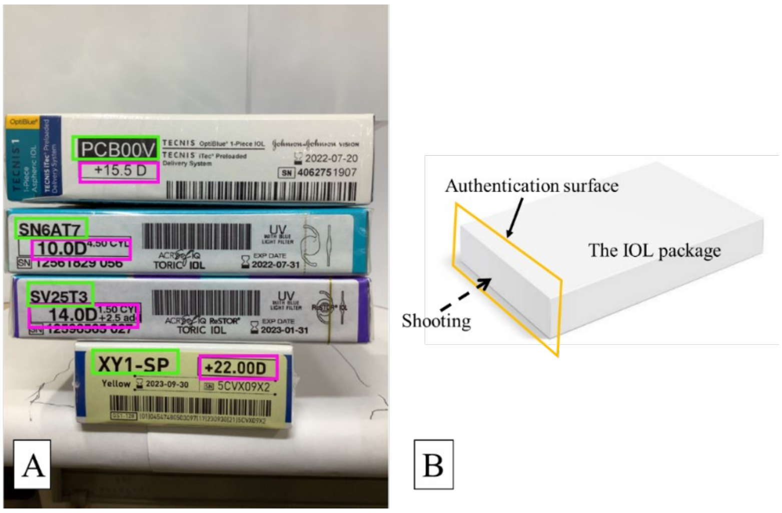

A, Example IOL authentication photograph. The violet boxes indicate the power values of the IOLs. The green boxes indicate the names of the IOLs. Although this example shows four IOLs, the system can identify up to 6 different IOL models stacked in a similar manner. B, the surface to be photographed for IOL authentication (yellow-line area) is often on the short side of the package. A dotted arrow indicates the direction from which the photograph is taken.

It should be noted that the locations of the IOL model name and power value on the authentication area are not fixed. Furthermore, the spacing and type faces of the numbers and letters vary slightly, even for the same model number. (C and D) Therefore, it was necessary to create image datasets reflecting these variations by augmentation for the model to learn a wide variety of patterns. Iterative training and validation using the produced data improved the model performance.

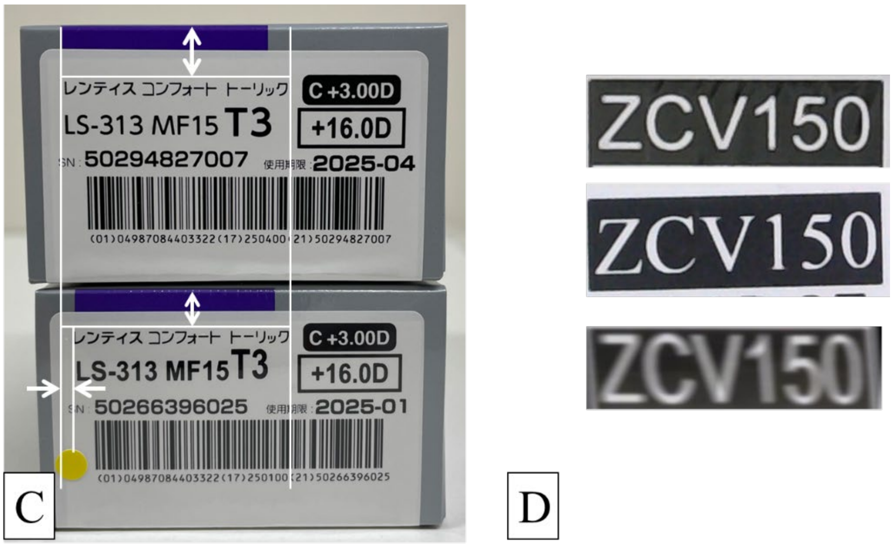

C. Photographs of the same lens type with the same power are arranged vertically. The straight white lines and arrows indicate the displacement between the two boxes where the same content is printed.

D. Enlarged image of the printed area of the same lens type name from the three certified photographs. These three photographs show that the fonts used are different, even though they are the same. And we can see skewed or out-of-focused photography.

Supplemental Figure 6

AI System Performance in Authentication Tasks: 100% Sensitivity and Specificity

Given the low incidence of medical errors in real-world settings, evaluating the error identification performance of the AI-based Surgery Safety System is challenging. To address this, we created a dataset of 300 cases, with 50% containing intentional errors, as shown in the figures below:

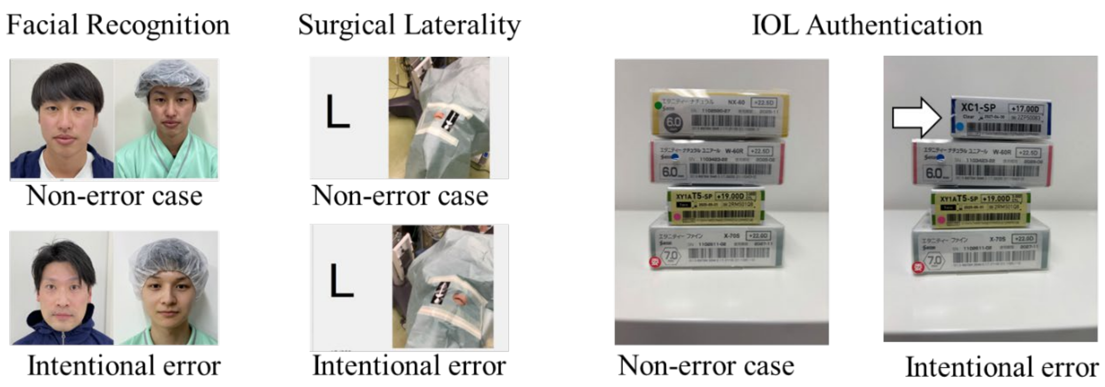

In the Facial Recognition test: The upper row shows a correct case where the reference photo (left) and the pre-surgery photo (right) are of the same person. The lower row shows an intentional error case where the photos are of different individuals.
For Surgical Laterality: The upper row correctly shows the surgical drape opening on the left side. The lower row presents an intentional error with the opening on the right side.
In the IOL Authentication test: Four types of lenses were set up separately. The left side shows a non-error case where the lenses match the reference master data. The right side presents an intentional error case with a deliberately mismatched lens (indicated by the white arrow).

As shown in the graph below, the AI system made no mistakes in identifying both correct cases and intentional errors:

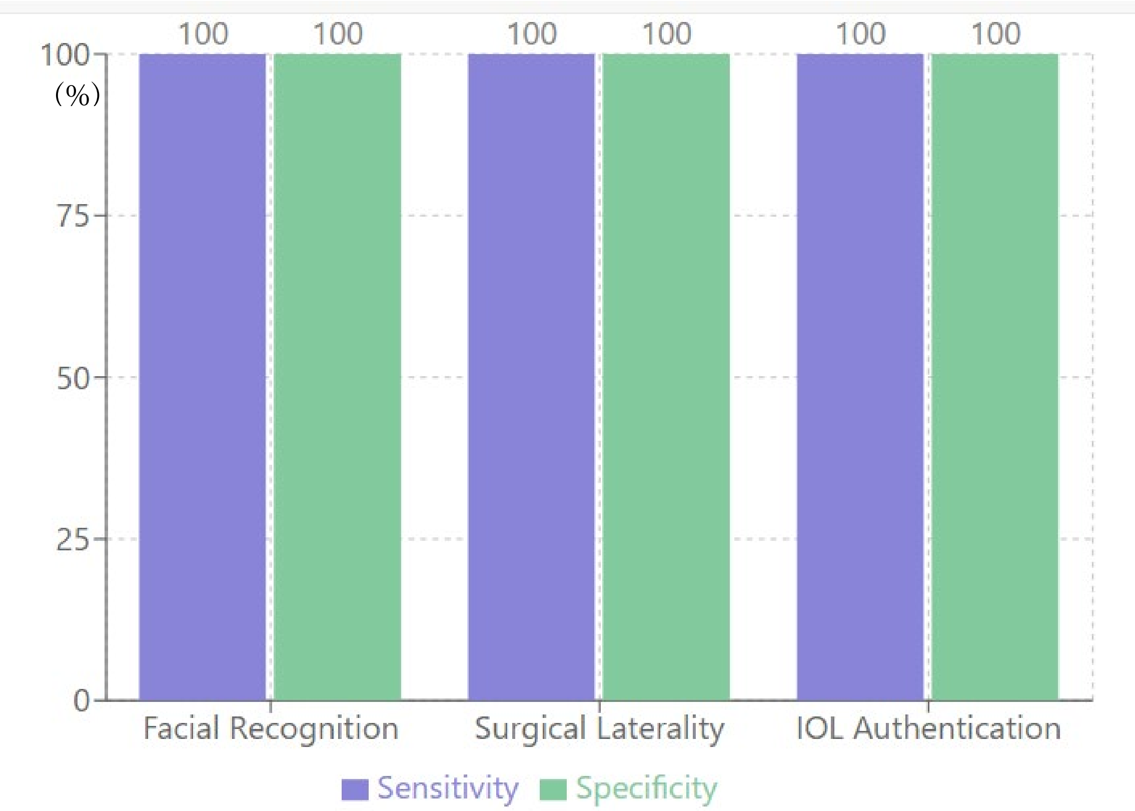

Blue bars: Sensitivity - Correct identification of intentional errors (true positive rate)

Green bars: Specificity - Correct identification of non-error cases (true negative rate)

This graph represents the performance of the AI system in three authentication tasks: Facial Recognition, Surgical Laterality, and IOL Authentication. The study involved a total of 300 cases, equally divided between intentional errors (150) and correct cases (150).

Sensitivity measures the system's ability to correctly identify all intentional errors, while specificity indicates its ability to correctly identify all non-error cases. In all three authentication types, the AI system achieved 100% accuracy for both sensitivity and specificity, demonstrating perfect performance in distinguishing between error and non-error cases.

Supplemental Figure 7

Alert Screen Prompting Re-Shooting Due to Low Quality of Authentication Image

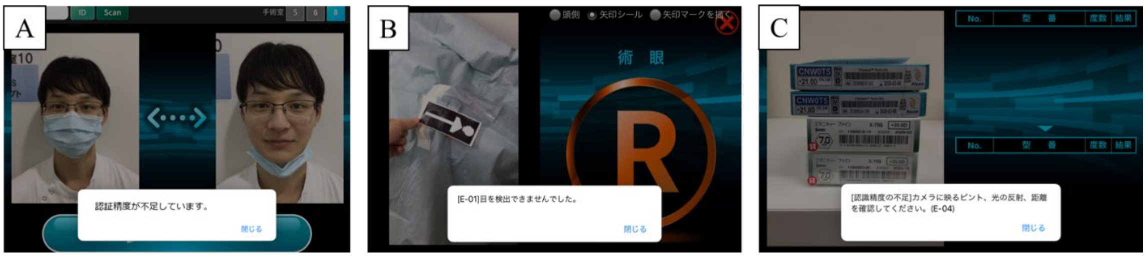

A: Facial Recognition: "Authentication accuracy is insufficient." B: Surgical laterality: "Unable to detect the eye." C: IOL (Intra Ocular Lens) Recognition: "Authentication accuracy is insufficient. Check the focus, light reflection, and distance captured by the camera.

Supplemental Figure 8

Examples of Authentication Failures Due to Poor Quality of Authentication Photos

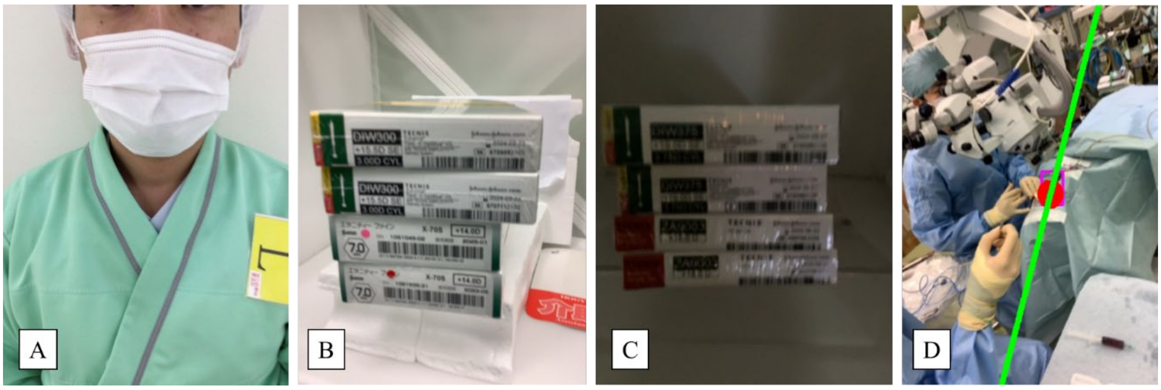

These are all examples where authentication failed because the quality of the authentication photos was low. Despite the system internally determining this and prompting for retakes, authentication ultimately failed.

A: In facial authentication, the camera angle is poor, with the face barely visible in the frame. (These photos depict a simulated patient scenario, recreated by a co-author, to precisely match the conditions of actual authentication photos.)

B: During intraocular lens (IOL) authentication, the photo is out of focus, making it difficult to identify the numbers printed on the IOL case.

C: During IOL authentication, there is insufficient background lighting, making the image too dark for authentication.

D: During Surgical laterality authentication, the physician was uncooperative. By the time the authentication photo was taken, the surgery had already begun, and the surgeon's fingers were obscuring the opening in the surgical drape, making authentication impossible.

Supplemental Figure 9

Authentication process and definition of terms

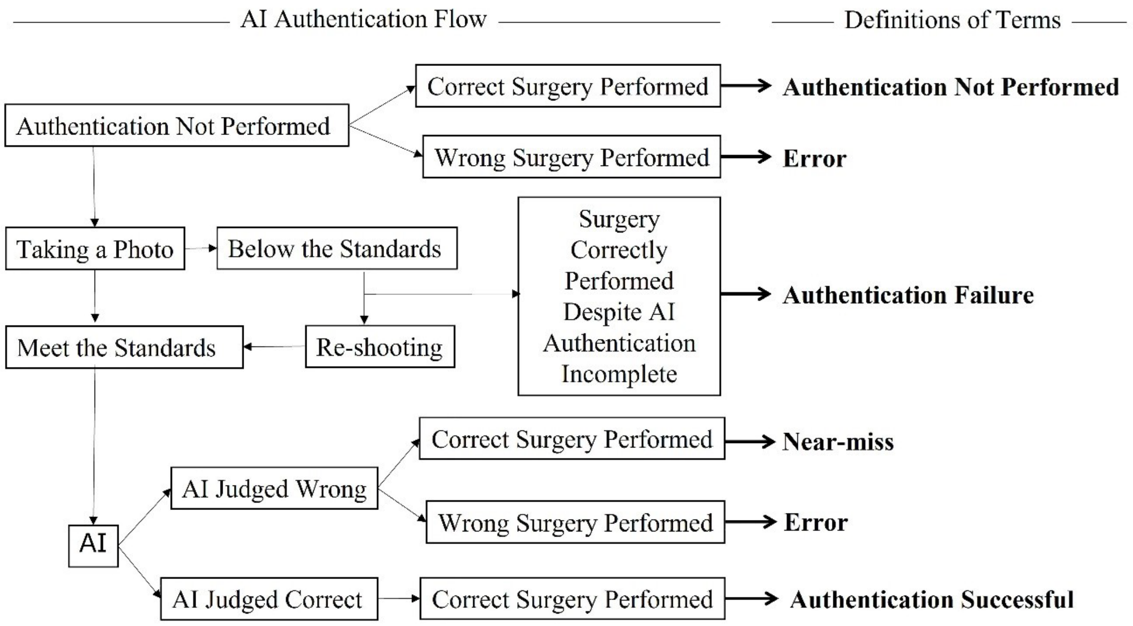

Definitions of authentication patterns are established following the authentication flow of AI-based Surgery Safety. While there are other patterns that could theoretically occur, all patterns obtained in this retrospective study are covered here

Supplemental Figure 10

The process of IOL data entry into the reference database, which includes multiple verification steps to prevent transcription errors.

Surgical data, right-or-left eyes to receive operation, planned operative method, IOL model name, powers of the IOL are registered to the reference database. Both powers of the IOL in the bag and on the sulcus were calculated by ophthalmologists using the required three anatomical measurements (axial length and both flatter and steeper corneal curvatures), targeted focus, the A constant of IOL, and SRK/T formula [4] built-in the reference database. (A and B) The doctor selected the IOL power in the reference database by visually comparing the result of the reference database's SRK/T and other formulas calculated on the IOL master 700 (ZEISS USA), an ocular axis length measuring device. (C and D) This recalculation and selection method on the electronic monitor prevents simple data transcription errors. Besides one ophthalmologist and two optometrists reviewed the registered data seeking for mistyping or incoherence among the registered data.

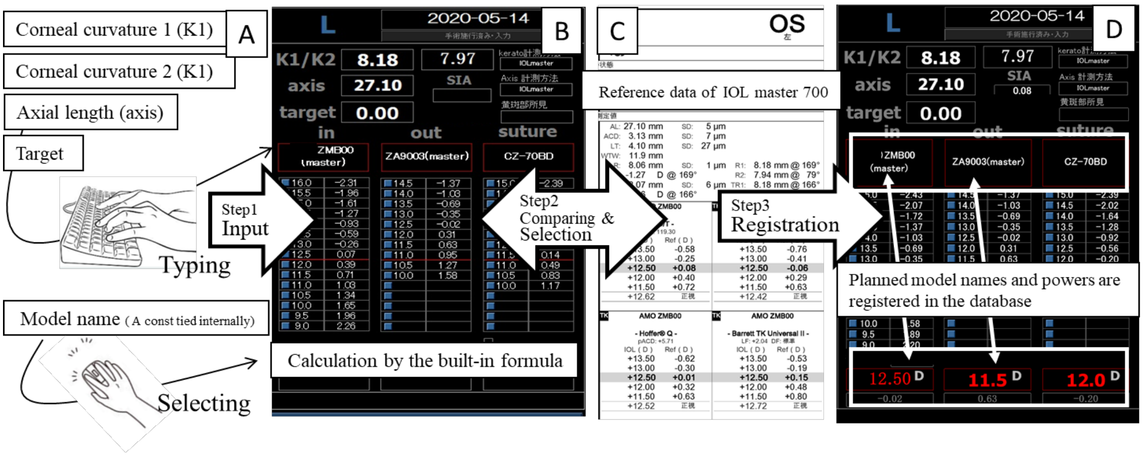

A: To calculate the optimal IOL power, doctors type required 4 values and select the IOL model name from pulldown menu provided by the reference database. B: A constant of IOL and SRK/T formula built-in the reference database provides optimal IOL powers. C: The doctor selected the IOL power in the reference database by visually comparing with other formulas calculated on the IOL master 700 (ZEISS USA), an ocular axis length measuring device. D: Planned IOL model names and powers are registered in the reference database electronically without using handwriting.

Supplemental Figure 11

The data entry and verification process

The master data referenced by AI-based Surgery Safety is registered in the surgical management system. All utilities related to surgical information, such as the daily surgery list or the individual specific IOL list (A and B), are automatically generated based on the surgical management system. Every professional, including doctors, nurses, orthoptists, and engineers, relies on this system's information when needed, for tasks like planning the order of surgeries and explaining procedures to patients. This process also serves as a check to ensure that the input data is correct.

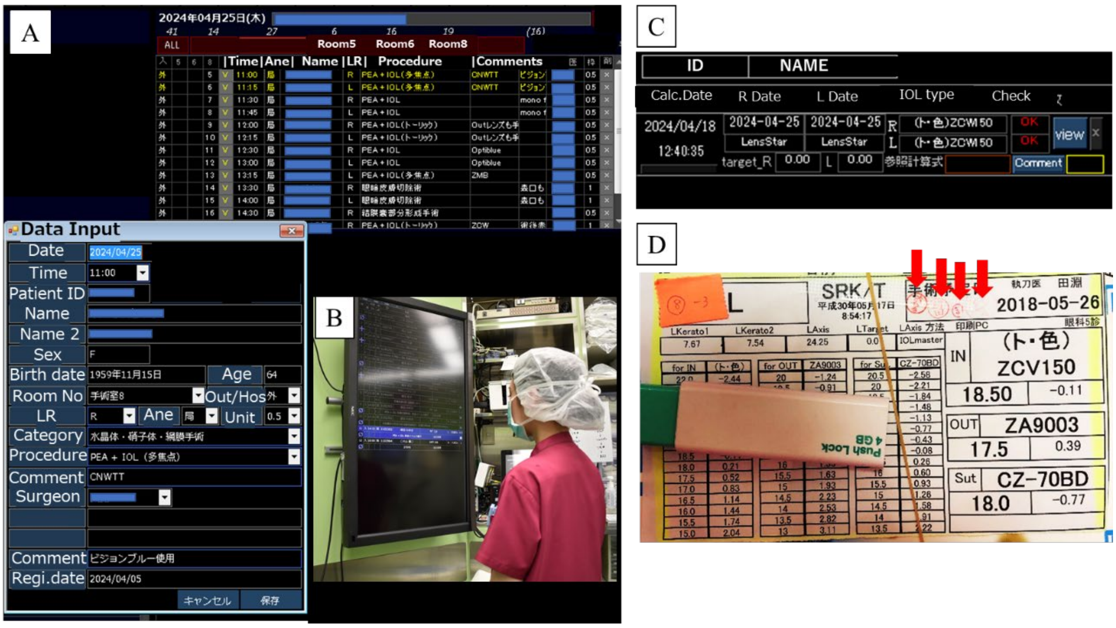

A: The upper right portion displays the day's surgery list, with tabs allowing for viewing by individual operating rooms or all rooms together. This list also serves as a handy reference sheet for nurses. The lower left portion contains a pop-up screen for entering surgery information, including details such as surgery date, time, patient ID, name (in kanji), name 2 (in hiragana), gender, birth date, left/right designation, surgery category, procedure, remarks, operating surgeon, outpatient/inpatient classification, surgery time unit (in 30-minute increments), and anesthesia type (local or general), among other details.

B: The surgical list monitor in each operating room continuously displays the status of ongoing, waiting, and completed surgeries, allowing medical professionals to monitor the real-time situation. This monitoring system also provides feedback, especially in cases of incorrect data entry.

C: The patient-specific IOL registration screen is linked to the surgical database by ID, name, surgery date, and left/right designation, recording the type and power of the IOL to be inserted.

D: IOL registration data is used for the IOL administration sheet. Four red arrows indicate stamp marks showing that four checks were performed by certified orthoptists. This process also helps prevent the wrong preparation of IOLs.

Supplemental Figure 12

Sequence of AI Authentication Attempts in a Case of Wrong-Side Anesthesia Administration

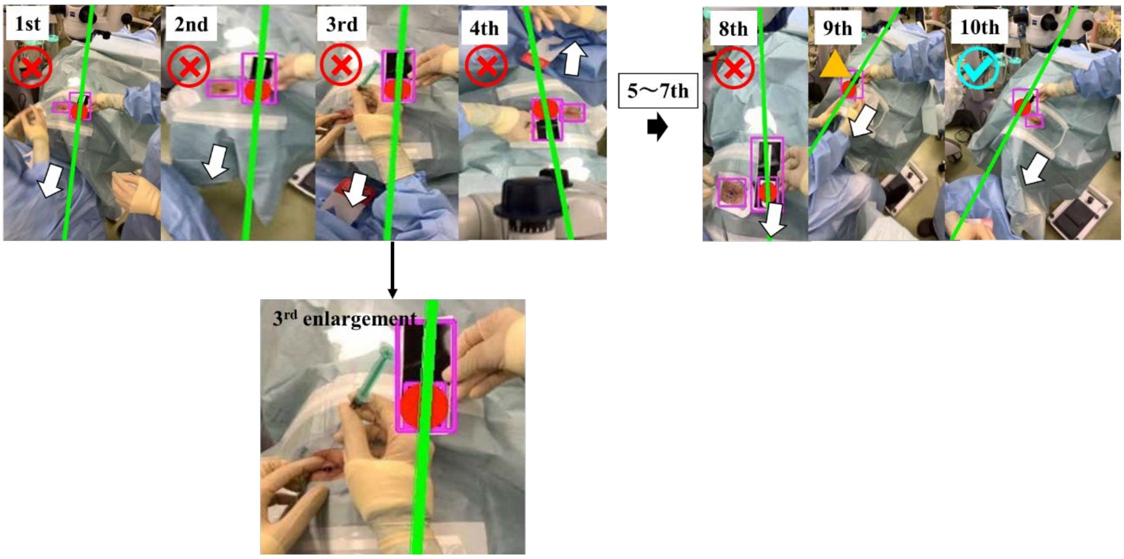

This figure illustrates a series of AI authentication attempts during an ophthalmic surgical procedure where wrong-side anesthesia administration occurred despite both AI and WHO checklist implementation. In the AI authentication results, red cross marks indicate incorrect side detection, yellow triangles signify poor image quality requiring retake, and the blue checkmark confirms successful authentication. White arrows indicate the direction towards the patient's head. Image1-5 and 8 show wrong side draping detected by the AI (red cross mark) Images 1-3 show correct camera positioning from the head side. Image 4 reveals a shift in camera angle to the foot end, potentially causing confusion for the nursing staff about the source of authentication failure. The 3rd image enlargement demonstrates the moment of incorrect anesthesia administration to the contralateral eye. Images 5-7 are omitted for brevity. Image 8 shows the realization of incorrectly placed surgical drapes. Images 9 and 10 depict attempts at correct authentication, with image 9 failing due to poor image quality (yellow triangle) and image 10 finally succeeding (blue checkmark).

This sequence highlights potential communication breakdowns and cognitive biases in interpreting AI system feedback, emphasizing the importance of clear protocols and human factors in AI-assisted surgical safety systems.

Supplemental Figure 13

AI Authentication Process in Preventing Intraocular Lens and Surgical Draping Errors

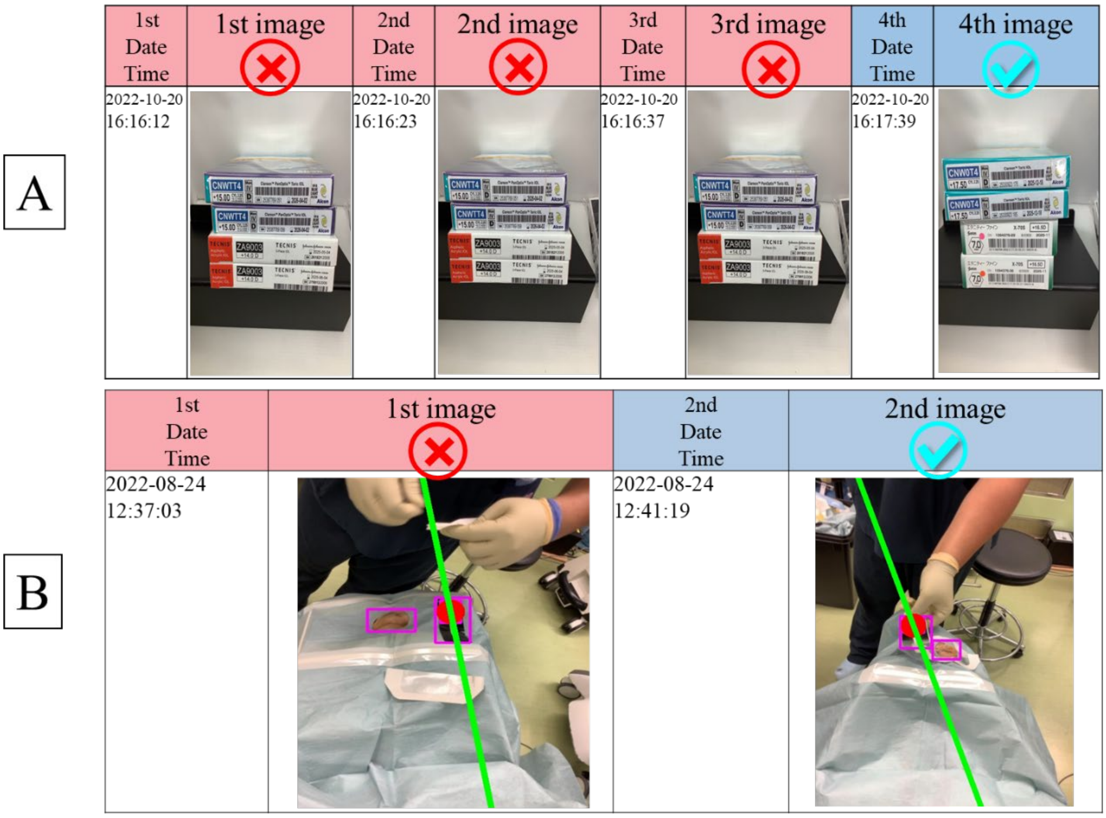

This supplementary figure illustrates two critical scenarios where the AI-based authentication system played a crucial role in preventing surgical errors.

Panel A demonstrates a near-miss in intraocular lens (IOL) preparation. The first three authentication attempts (at 16:16:12, 16:16:23, and 16:16:37) show incorrect IOLs intended for a different operating room, as indicated by the red cross marks. After the third attempt, an attentive nurse not assigned to the case identified the error. The fourth image (at 16:17:39) shows the correct IOLs being successfully authenticated, as indicated by the blue checkmark.

Panel B illustrates a case of incorrect surgical draping for an anti-VEGF intravitreal injection. The procedure was planned for the left eye. The first image (at 12:37:03) reveals incorrect draping with the right eye exposed, as shown by the red cross mark. The second image (at 12:41:19) displays the corrected draping with the left eye properly exposed, confirmed by the blue checkmark.

These examples highlight the effectiveness of the AI authentication system in detecting and preventing potential surgical errors, emphasizing its role in enhancing patient safety in ophthalmic procedures. The system's ability to identify discrepancies in both surgical equipment and patient preparation demonstrates its comprehensive approach to error prevention. Furthermore, the AI verification images being stored in the system are very useful for feedback to improve surgical safety. These stored images provide valuable data for retrospective analysis, training, and continuous improvement of both the AI system and human protocols.

Supplemental Figure 14

Monthly Trend of IOL Authentication Rate and Unlearned IOL Usage Ratio

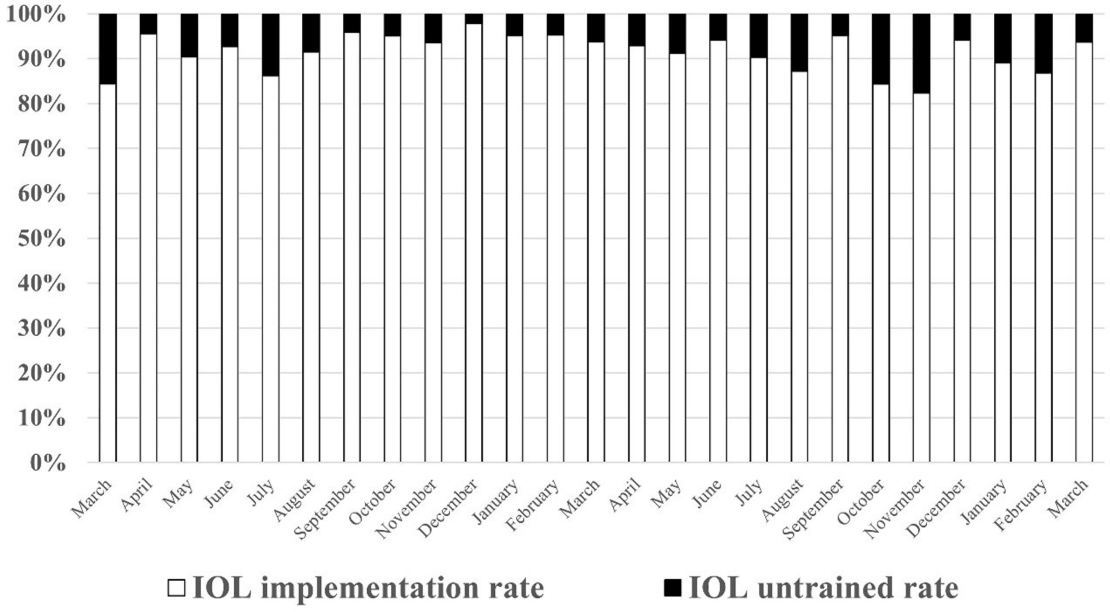

The white area of the bar graph indicates the IOL authentication rate, while the black area shows the ratio of unlearned IOLs. An average of 7.3% of IOLs used during the period were unlearned.

Supplemental Table 1

Calculation process of cost benefit analysis.

Category	Item	Unit cost	N	Category cost	Detail
**Cost**					
System Implementation Cost	Hardware costs (e.g., iPads, servers, etc.)	US$28,968.00	1	US$20,889.60	Tsukazaki Hospital expense data
	Software development, AI model training and implementation, AI model update and retraining costs	US$424,408.40	1	(US$424,408.40)	Tsukazaki Hospital expense data
	System integration costs	US$5,440.00	1	(US$5,440.00)	Tsukazaki Hospital expense data
	Human resources cost of IOL AI verification	US$0.62	9713	(US$5,973.50)	Average authentication time for face, IOL, RL: 11.8s, 8.57s, 3.1s respectively. Reference face photo time: 30s.

					Nurse hourly wage: $41 [5]
	Human resources cost of other surgery AI verification	US$0.51	9049	(US$4,614.99)	Average authentication time for face, RL: 11.8s, 3.1s respectively. Reference photo time: 30s. Nurse hourly wage: $41 [5]
Operational Cost	Maintenance costs (hardware, software)	US$482.80	25	(US$12,070.00)	Calculated as monthly cost for 25 months, assuming 5-year renewal for equipment
	Additional personnel costs (e.g., system administrators), Training costs (for medical staff)	US$272.00	50	(US$13,600.00)	Bi-monthly 1-hour operation meeting (1 each of doctor, nurse, optometrist, engineer). Engineer cost included in operational cost [5-7]
Traditional Safety Measures Cost	WHO surgical safety checklist implementation and Personnel costs related to traditional safety measures IOL	US$8.98	9713	(US$87,222.74)	For IOL surgery: 10s optometrist check x 4 people = (10/3600) x 66 x 4 = 0.73. Doctor's 3-min check = 165 x 3/60 = 8.25. Total US$8.98 [6,7]
	WHO surgical safety checklist implementation and Personnel costs related to traditional safety measures Every surgery	US$2.06	18762	(US$38,649.72)	Nurse's left/right label, ID band, notation check: 1 min each, total 3 min = (3/60) *41=2.05. Timeout 30s (1 Dr, 2 Ns) = (30/3600) *(165+2*41) =2.06 [5,6]
**Benefit**					
Medical Error Related Cost	Additional treatment costs due to medical errors (IOL)	US$5,340.50	n1	n1 X US$5,340.5	Cataract surgery
	Additional treatment costs due to medical errors (Surgical laterality)	US$1,925.00	n2	n2 X US$1,925.0	Assumed as anti-VEGF intravitreal injection (as all left/right errors in this study were injection procedures)
	Estimated legal compensation amounts Wrong IOL	US$90,000.00	n1	n1 X US$90,000	Based on U.S. litigation cases [8]
	Estimated legal compensation amounts Wrong Side	US$136,452.84	n2	n2 X US$136,452	Based on U.S. Surgical laterality litigation cases [9]

Legal and Defense Costs	US$27,000.00	n1+n2	(n1+n2) X US$27,000	Based on per-case legal costs in U.S. medical litigation [10]
Estimated revenue impact due to hospital reputation decline	US$44,400.00	n1+n2	(n1+n2) X US$44,400	Cost of reputation decline calculated as compensation won by hospitals for defamation [11]

Notes:

CBA: Cost-Benefit Analysis

Most conservative setting (excluding near-miss cases, assuming prevention of 2 IOL errors and 2 wrong-side errors that occurred without AI use): US$181,946.94

Intermediate setting (assuming 50% of near-misses would become errors, preventing 15 IOL errors and 4 wrong-side errors): US$2,769,129.12

Highest risk setting (assuming all near-miss cases would become errors, preventing 28 IOL errors and 6 wrong-side errors): US$5,356,311.30

Salary (Annual / Hourly):

Ophthalmologist: US$329,644.00 / US$165.00 [6]

Nurse: US$82,750.00 / US$41.00 [5]

Optometrist: US$132,524.00 / US$66.00 [7]

Surgical Fees:

Cataract surgery cost (USA) [12]: US$5,340.50

Anti-VEGF intravitreal injection cost (USA) [13]: US$1,925.00

Supplemental Table 2

The numbers for each outcome definition

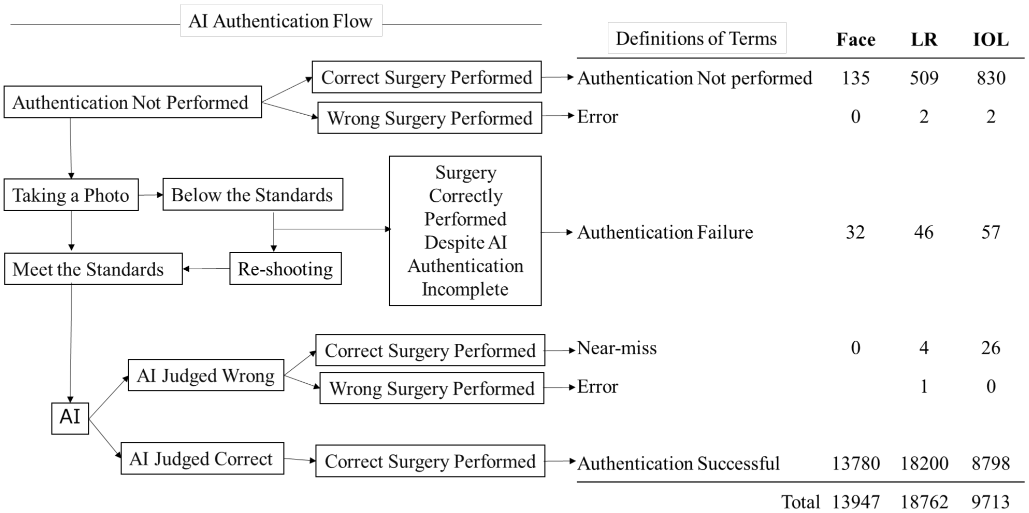

Definitions of Terms	Face	LR	IOL
Authentication Not performed	135	509	830
Error	0	2	2
Authentication Failure	32	46	57
Near-miss	0	4	26
Error		1	0
Authentication Successful	13780	18200	8798
Total	13947	18762	9713

Supplemental Table 3

Implementation rates and ineligible cases.

	Overall	In eligible cases	In eligible cases excluding initial 3 months	in eligible cases of initial 3 months	Total number of ineligible cases (below columns)	(Untrained cases)	(Unequipped Room)	(System down)
**IOL implementation rate**	91.5 %	98.7%	99.2%	95.4%	715	711	0	4
**Surgical laterality implementation rate**	97.3 %	97.5%	99.3%	84.5%	42	0	33	9
**Face implementation rate**	99.0 %	99.2%	99.3%	98.8%	28	0	22	6
**Total implementation rate**	95.9 %	98.3%	99.3%	91.8%	785	711	33	13

Detailed Technical Specifications and Analyses

1. Technical Details of AI Components

a) Facial Recognition System

- Component: ISG-539 face recognition development kit (Glory, Himeji, Hyogo, Japan)
- Performance: False positive rate < 1/100,000
- Matching criteria: 75% match between authentication and reference photographs
- COVID-19 adaptation: Enhanced model to accommodate mask-wearing
- Technical implementation: Deep learning-based model with real-time processing capability

b) Surgical Laterality Verification

- Architecture: Two-stage object detection model using YOLOv3
- Function: Identifies positional relationship between surgical drape opening and non-operative side marker
- Model parameters:
  - First stage: momentum=0.9, decay=0.000001, learning rate=0.001
  - Training dataset: 1,171 images for initial stage
  - Second stage: 1,167 images with identical parameters
- Validation approach: Real-time verification with spatial relationship analysis

c) IOL Authentication System

- Three-stage process:
  1. Authentication area identification (YOLOv3)
  2. IOL model number recognition (VGG16)
  3. IOL power verification (VGG16)
- Training specifications:
  - Authentication area: 1,732 images

  - Model number recognition: 123,616 images (136 IOL models)
  - Power verification: 57,126 images (121 power variations)
- Simultaneous processing capability: Up to 6 stacked IOL boxes

2. Performance Metrics Details

a) Authentication Attempts and Time Analysis

For each authentication type, we evaluated the mean number of attempts required for successful authentication and the average time taken:

Facial Recognition:

Mean Attempts: 1.13 (95% CI: 1.10–1.16)

Average Time: 11.8 seconds (95% CI: 2.6–21.0 seconds)

Surgical Laterality Recognition:

Mean Attempts: 1.05 (95% CI: 1.03–1.08)

Average Time: 3.10 seconds (95% CI: 1.45–4.75 seconds)

Intraocular Lens (IOL) Authentication:

Mean Attempts: 1.15 (95% CI: 1.10–1.20)

Average Time: 8.57 seconds (95% CI: 5.84–11.3 seconds)

b) Unlearned IOLs

Incidence of Unlearned Lenses: Out of 9,713 IOL surgeries performed during the post-implementation period, 7.3% (95% CI: 6.8%–7.8%) involved unlearned lenses that were not recognized by the AI system.

Incorrect IOL Implantations: Two cases of incorrect IOL implantation were attributed to these unauthenticated lenses.

Monthly Trend: The monthly ratio of unlearned lenses is depicted in Supplemental Figure 14, showing fluctuations over the implementation period.

c) Authentication Failures Due to Substandard Image Quality

We defined authentication failures as instances where surgery proceeded despite the AI system's inability to authenticate due to poor image quality:

Facial Recognition:

Failure Rate: 0.25% (33 out of 13,250 cases; 95% CI: 0.17%–0.35%)

Surgical Laterality Recognition:

Failure Rate: 0.24% (42 out of 17,409 cases; 95% CI: 0.17%–0.32%)

IOL Authentication:

Failure Rate: 0.66% (56 out of 8,526 cases; 95% CI: 0.50%–0.85%))

3. Detailed Economic Analyses

a) Net Present Value (NPV) Calculations

We conducted a Net Present Value analysis over a five-year period using a 3% annual discount rate to compare the economic feasibility of the AI-based Surgery Safety system with traditional safety measures.

Total Costs Over Five Years:

AI System: $461,426.49

Traditional Safety Measures: $125,872.46

Benefit Calculations Across Different Risk Settings:

Conservative Setting (only prevented errors considered as benefits):

Total Benefits: $181,946.94

NPV: -$293,566.68

Intermediate Setting (assuming 50% of near-misses would result in errors):

Total Benefits: $2,769,129.12

NPV: $2,093,468.31 (95% CI: $1,519,215.03 to $2,667,721.59)

High-Risk Setting (all near-misses treated as potential errors):

Total Benefits: $5,356,311.30

NPV: $4,480,503.30

b) Cost-Effectiveness Analysis (CEA)

We performed a Cost-Effectiveness Analysis to determine the Incremental Cost-Effectiveness Ratio (ICER) of implementing the AI system compared to traditional safety measures.

Errors Prevented:

Conservative Scenario: 4 errors prevented

Intermediate Scenario: 19 errors prevented

High-Risk Scenario: 34 errors prevented

ICER Calculations:

Conservative Scenario:

ICER: $111,851.34 per additional error prevented

Intermediate Scenario:

ICER: $18,641.89 per additional error prevented

High-Risk Scenario:

ICER: $10,167.21 per additional error prevented

c) Sensitivity Analysis

A sensitivity analysis was conducted to assess the robustness of the economic outcomes by varying the medical error rates by ±20% in the intermediate setting.

Variation in Medical Error Rates:

-20% Error Rate:

NPV: $1,674,774.65

+20% Error Rate:

NPV: $2,512,161.97

This analysis indicates that even with fluctuations in error rates, the AI system maintains a positive NPV, underscoring its economic viability across a range of scenarios.

d) Summary of Economic Findings

Cost Comparison: The AI system incurs higher initial and operational costs ($461,426.49) compared to traditional safety measures ($125,872.46) over five years.

Economic Benefits: In the intermediate setting, the AI system yields a positive NPV of $2,093,468.31, suggesting that the benefits outweigh the costs significantly.

Cost-Effectiveness: The ICER decreases markedly from the conservative to the high-risk scenario, indicating improved cost-effectiveness as the number of errors prevented increases.

Robustness of Results: Sensitivity analysis confirms that the economic advantage of the AI system persists even when medical error rates vary by ±20%.

Reference

1. Arrieta AB, Díaz-Rodríguez N, del Ser J, Bennetot A, Tabik S, Barbado A, et al. Explainable Artificial Intelligence (XAI): Concepts, taxonomies, opportunities and challenges toward responsible AI. Information Fusion 2020;58:82–115.

2. van der Ham IJ, Dijkerman HC, van Stralen HE. Distinguishing left from right: A large-scale investigation of left–right confusion in healthy individuals. Q J Exp Psychol (Hove) 2021;74(3):497–509.

3. Simonyan K, Zisserman A. Very Deep Convolutional Networks for Large-Scale Image Recognition. arXiv preprint arXiv:1409.1556 [cs.CV]; 2014.

4. Karabela Y, Eliacik M, Kaya F. Performance of the SRK/T formula using A-scan ultrasound biometry after phacoemulsification in eyes with short and long axial lengths. BMC Ophthalmol 2016;16:96.

5. Teach.com. Nursing Salary by State. Teach.com. https://teach.com/online-ed/healthcare-degrees/online-msn-programs/nursing-salary-by-state/. Accessed 31 Aug 2023.

6. Salary.com. Ophthalmologist Salary. Salary.com. https://www.salary.com/research/salary/benchmark/ophthalmologist-salary. Accessed 31 Aug

2023.

7. Your Total Vision. How Much Do Optometrists Make with Their Own Practice? Your Total Vision. https://yourtotalvision.com/how-much-do-optometrists-make-with-their-own-practice/. Accessed 31 Aug 2023.

8. Kim JE, Weber P, Szabo A. Medical malpractice claims related to cataract surgery complicated by retained lens fragments (an American Ophthalmological Society thesis). Trans Am Ophthalmol Soc 2012;110:94–116.

9. Tan J, Ross JM, Wright D, Gupta A, Tarchini G, Suarez S, et al. A contemporary analysis of closed claims related to wrong-site surgery. Jt Comm J Qual Patient Saf 2023;49(5):265–273.

10. Carroll AE, Parikh PD, Buddenbaum JL. The impact of defense expenses in medical malpractice claims. J Law Med Ethics 2012;40(1):135–42.

11. Carruthers Law. Damages Awards in Defamation Claims. Carruthers Law. https://www.carruthers-law.co.uk/our-services/defamation/damages-awards-in-defamation-claims/. Accessed 31 Aug 2023.

12. All About Vision. How Much Does Cataract Surgery Cost? All About Vision. https://www.allaboutvision.com/conditions/cataract-surgery-cost.htm. Accessed 31 Aug 2023.

13. WebMD. Wet AMD Treatment Costs. WebMD. https://www.webmd.com/eye-health/macular-degeneration/wet-amd-treatment-costs. Accessed 31 Aug 2023.
